# Supplementary material for: Monitoring the after-effects of ischemic stroke through EEG microstates
Source: PLoS One. 2024 Mar 22;19(3):e0300806. doi: 10.1371/journal.pone.0300806 (PMC10959352; doi:10.1371/journal.pone.0300806)
Supplement: Attachment — Submitted filename: Response to Reviews.pdf [file pone.0300806.s002.pdf]

Response to the Review

Manuscript Ref No.: PONE-D-23-05197

"Monitoring the after-effects of Ischemic Stroke through EEG Microstates"

Fang Wang, Xue Yang, Xueying Zhang, Fengyun Hu

First of all, we would like to acknowledge the editor, associate editor and anonymous reviewers for their comments and suggestions. These comments and suggestions are invaluable for us to improve the quality and readability of our manuscript. We have considered these comments and suggestions carefully, and then have revised the paper accordingly. Following the Editor's suggestions, we now resubmit our revised manuscript for your further consideration for publication.

The followings are our detailed responses to the editor and reviewers on how we have addressed all their comments and suggestions and what changes we have made accordingly to the original version of our manuscript. All changes are marked in red in our revised manuscript.

---

1 Response to Editor-in-Chief

a) The entire editorial decision letter:

Dear Dr. Wang,

Thank you for submitting your manuscript to PLOS ONE. After careful consideration, we feel that it has merit but does not fully meet PLOS ONE's publication criteria as it currently stands. Therefore, we invite you to submit a revised version of the manuscript that addresses the points raised during the review process.

Please submit your revised manuscript by May 12 2023 11:59PM. If you will need more time than this to complete your revisions, please reply to this message or contact the journal office at plosone@plos.org. When you're ready to submit your revision, log on https://www.editorialmanager.com/pone/and select the 'Submissions Needing Revision' folder to locate your manuscript file.

Please include the following items when submitting your revised manuscript: A rebuttal letter that responds to each point raised by the academic editor and reviewer(s). You should upload this letter as a separate file labeled 'Response to Reviewers'. A marked-up copy of your manuscript that highlights changes made to the original version. You should upload this

as a separate file labeled 'Revised Manuscript with Track Changes'. An unmarked version of your revised paper without tracked changes. You should upload this as a separate file labeled 'Manuscript'. If you would like to make changes to your financial disclosure, please include your updated statement in your cover letter. Guidelines for resubmitting your figure files are available below the reviewer comments at the end of this letter.

If applicable, we recommend that you deposit your laboratory protocols in protocols.io to enhance the reproducibility of your results. Protocols.io assigns your protocol its own identifier (DOI) so that it can be cited independently in the future. For instructions see: https://journals.plos.org/plosone/s/submission-guidelinesloc-laboratory-protocols. Additionally, PLOS ONE offers an option for publishing peer-reviewed Lab Protocol articles, which describe protocols hosted on protocols.io. Read more information on sharing protocols at https://plos.org/protocols utm$_{m}edium = editorial - emailutm_{s}ource = authorlettersutm_{c}ampaign = protocols$.

We look forward to receiving your revised manuscript.

Kind regards,

Tommaso Martino, M.D. Academic Editor PLOS ONE

b) ***Our Response:*** *Thank you for your encouraging comments and suggestions. In our revised manuscript, we have addressed all comments from you and the three anonymous reviewers #1, #2 and #3. According to your request, we have submitted three files named "Response to Reviewers", "Revised Manuscript with Track Changes" and "Manuscript". This document includes our responses to the comments of reviewers in the following Section 2 of this document. Our point-to-point responses to each reviewer are respectively enclosed in Sections Sections 3, Sections 4 and Sections 5 of this document. Thank you.*

---

2 Response to Reviewers' comments

a) Reviewers' comments:

Reviewer's Responses to Questions

Comments to the Author

1. Is the manuscript technically sound, and do the data support the conclusions?

The manuscript must describe a technically sound piece of scientific research with data that supports the conclusions. Experiments must have been conducted rigorously, with appropriate controls, replication, and sample sizes. The conclusions must be drawn appropriately based on the data presented.

Reviewer 1: Yes

Reviewer 2: Yes

Reviewer 3: Yes

2. Has the statistical analysis been performed appropriately and rigorously?

Reviewer 1: Yes

Reviewer 2: Yes

Reviewer 3: Yes

3. Have the authors made all data underlying the findings in their manuscript fully available?

The PLOS Data policy requires authors to make all data underlying the findings described in their manuscript fully available without restriction, with rare exception (please refer to the Data Availability Statement in the manuscript PDF file). The data should be provided as part of the manuscript or its supporting information, or deposited to a public repository. For example, in addition to summary statistics, the data points behind means, medians and variance measures should be available. If there are restrictions on publicly sharing data—e.g. participant privacy or use of data from a third party—those must be specified.

Reviewer 1: No

Reviewer 2: Yes

Reviewer 3: No

4. Is the manuscript presented in an intelligible fashion and written in standard English?

PLOS ONE does not copyedit accepted manuscripts, so the language in submitted articles must be clear, correct, and unambiguous. Any typographical or grammatical errors should be corrected at revision, so please note any specific errors here.

Reviewer 1: Yes

Reviewer 2: Yes

Reviewer 3: No

5. Review Comments to the Author

Please use the space provided to explain your answers to the questions above. You may also include additional comments for the author, including concerns about dual publication, research ethics, or publication ethics. (Please upload your review as an attachment if it exceeds 20,000 characters)

b) Our Response: *Thanks the three reviewers comments. Among the first four questions above, two questions (Question 3 and Question 4) received negative evaluations from one or two of the reviewers.*

Regarding question 3, we were not able to provide all the data in the manuscript due to restrictions on publicly sharing EEG clinical data from the hospital and patients.

As for question 4, we have revised our manuscript and improved its English representation based on the suggestions of the reviewers. The specific revisions and improvements are outlined in the point-to-point response to the reviewers in the following section.

3 Response to Review #1

Overall Comment: The authors investigate the correlation between EEG microstates with the clinical states of stroke patients, such as the level of consciousness, the existence of motor disturbance, and the side of the motor disturbance. This manuscript requires extensive revision before being reconsidered, particularly in terms of the detailed and justification of the proposed method, as well as the completeness of the results and comparisons. Following are my concerns.

Our Response: *Thank you for your comments. Your suggestions help us improve our research and manuscript. Our responses to your comments and suggestions are given below in detail.*

Comment 3.1: The foremost concern is number of subjects are very few for experimentation and dataset ethical approval, statistical analysis, age description details are missing. Did you read studies where authors utilized 60+ subjects to prove subject-independent motor imagery classification? Please see "Towards the Development of Versatile Brain-Computer Interfaces" and "A New Framework for Automatic Detection of Motor and Mental Imagery EEG Signals for Robust BCI Systems. If it looks difficult for you to perform experiments with large number of subjects at least discussed the above-mentioned studies.

Our Response: *Thanks for your critical comments and suggestion. We have read the two studies you suggested. We have added descriptions about the limitation of the number of subjects in our manuscript and cited two studies in the section on Limation of the revised manuscript:*

> *Another limitation is the number of subjects, however previous studies show that for the clinical data, the subject number is acceptable [35,36].*

Comment 3.2: For EEG Classification, first step is denoising of signals, MSPCA plays vital role, which is a combination of PCA and wavelet. I recommend authors to use MSPCA in discussion, The details of MSPCA can be found in "Motor imagery BCI classification based on novel two-dimensional modelling in empirical wavelet transform", "Motor Imagery BCI Classification Based on Multivariate Variational Mode Decomposition", "15.Motor Imagery EEG Signals Decoding by Multivariate Empirical Wavelet Transform-Based Framework for Robust Brain–Computer Interface", " Alcoholic EEG signals recognition based on phase space dynamic and geometrical features"

Our Response: *Thanks for your suggestions. We have cited two of the previous studies about denoising of EEG signals in the section of EEG Data Acquisition and Pre-processing in our manuscript as below:*

> *The trials with any of these artefacts were removed [19,20].*

Comment 3.3: For EEG Classification, convolutional neural networks play vital role, thus details should be included in literature, "Alcoholic EEG signals recognition based on phase space dynamic and geometrical features".

Our Response: *Thanks for your suggestion. The deep learning method plays a vital role in EEG classification. Therefore, we will employ deep learning methods in our future study. We have revised the section of Limitation of our manuscript as follows:*

> *Our future study will explore EEG data with more electrodes and subjects using deep learning methods and other EEG features.*

Comment 3.4: Graphical features are one of the newest approaches for identifying underlying

patterns of EEG signals and details of these methods can significantly increase the readability. Please include discussion from following articles "Depression Detection Based on Geometrical Features Extracted from SODP Shape of EEG Signals and Binary PSO", "Recognizing seizure using Poincaré plot of EEG signals and graphical features in DWT domain".

Our Response: *Thanks for your comments and suggestions. Graphical features are one of the approaches and we will explore this method in our future study.*

Comment 3.5: Please provide the details of future direction and possible solutions to continue this topic.

Our Response: *Thanks for your suggestion. We have updated our future direction in the section of Limitation of our manuscript as below:*

> *Our future study will explore EEG data with more electrodes and subjects using deep learning methods and other EEG features.*

Comment 3.6: Finally, I suggest authors to sit with English native speaker to improve the writing of proposed work.

Our Response: *Thanks for your suggestions. We have asked a native speaker to help us improve our manuscript.*

4 Response to Review #2

Overall Comment: In the current article, the authors of this article designed a framework to automatically detect disorder of consciousness (DoC) in ischemic stroke patients using computer-aided examinations of the electroencephalogram (EEG) microstates. They extracted microstates maps and calculated their statistical parameters to input into classifiers, specifically an ensemble of support vector machines (EOSVM), to identify DoC. The dataset was imbalanced, with the minority of patients being DoC, so EOSVM was designed to address this issue. The experimental results showed that the EOSVM classifier achieved better performance than other classifiers, with an accuracy and F1-Score both higher than 89%, and improved sensitivity from lower than 60% to higher than 80%. The authors concluded that the EOSVM-aided DoC detection based on microstates parameters is useful to medical practitioners and can save resources that would otherwise be consumed in traditional clinical checks. I want to say thank to authors for their great efforts on the submitted article. It seems that article is good and is ready to publish, but I just have some recommendations for authors:

Our Response: *Thanks for your encouraging comments. Our point-to-point responses to your comments and suggestions are given below in detail.*

Comment 4.1: reference are ok, but is better some of them be updated.

Our Response: *Thanks for your critical comments. We have added some latest research references in our manuscript below:*

> *The trials with any of these artefacts were removed [19,20].*
>
> *Another limitation is the number of subjects, however previous studies show that for the clinical data, the subject number is acceptable [35,36].*

Comment 4.2: the quality of figures are good, but it's better to show fig 1 and 2 in a better way.

Our Response: *Thanks for your suggestion. We have adjusted the display ratio of Fig 1 and Fig 2 to make them display in a better way.*

Comment 4.3: contribution section is missing in the introduction.

Our Response: *Thanks for your suggestion. We have added the contribution section in the introduction of the manuscript as below:*

Our work in this paper makes two main contributions:

1. *The first contribution is our establishment of correlations between EEG microstates with the clinical states of stroke patients through experimental studies of 152 patients. We designed an experimental procedure to extract microstate maps from a single dataset aggregated from multiple EEG datasets of all patients. Then, we investigated the correlations between EEG microstates with the level of DOC (awake, somnolence, stupor, light coma, middle coma, and deep coma) through the Spearman correlation coefficient. Moreover, an one-way ANOVA analysis was carried out to investigate the differences of EEG microstate parameters between different motor disturbance groups.*

2. *The second contribution is our design of a classifier for the detection of the DoC of stroke patients. We employed an ensemble of support vector machines (EOSVM) as the framework of the classifier. EEG microstates together with the statistical microstate parameters were input to the classifier. We compared the performance of the EOSVM under different numbers of microstate maps and different settings of the EOSVM majority voting.*

Comment 4.4: please say clearly in introduction the why you are using this method.

Our Response: *Thanks for your suggestion. Accepting your suggestion, we have added descriptions in the introduction to show why we use the EEG microstate method. The added part in the manuscript is as below:*

> *These previous studies have shown the potential of EEG microstates to extract characteristics from patients with stroke.*

Comment 4.5: please see these 3 articles and see how they addressed the results to the problems, I sure am it will help you a lot to improve your manuscript: A) Defending Smart Electrical Power Grids against Cyberattacks with Deep Q-Learning B) Model-free tracking control of regular and chaotic trajectories with machine learning C) Detecting Weak Physical Signal from Noise: A Machine-Learning Approach with Applications to Magnetic-Anomaly-Guided Navigation D) Adaptive optimal control of unknown discrete-time linear systems with guaranteed prescribed degree of stability using reinforcement learning

Our Response: *Thanks for your suggestion. We have read these four studies and they are really helpful for us to know how to address the results better. Therefore, we have added some descriptions figures in the classification section of the manuscript as below:*

> **Classification Results from other existing classifiers**
>
> *To further explore the performance of existing classifiers on our dataset, we used the other four classifiers: DTC, KNC, BC, and RF, to classify subjects with DoC and without DoC.*
>
> *To ensure stable classification operations, we used the stratified 5-fold cross-validation method to train the existing classifiers. This method, widely used in classification, ensures that the proportions between classes are the same in each fold as they are in the whole dataset.*

The 5-fold cross-validation classification results from the existing classifiers are shown in Figure 3. From the figure, it can be seen that the mean accuracy and F1-Score from the six classifiers are all below 80%. Although the mean specificity is good, almost 90%, the means of sensitivity from all six classifiers are below 65%. Sensitivity representing how many subjects with DoC are predicted correctly is the most critical evaluation metric in this study.

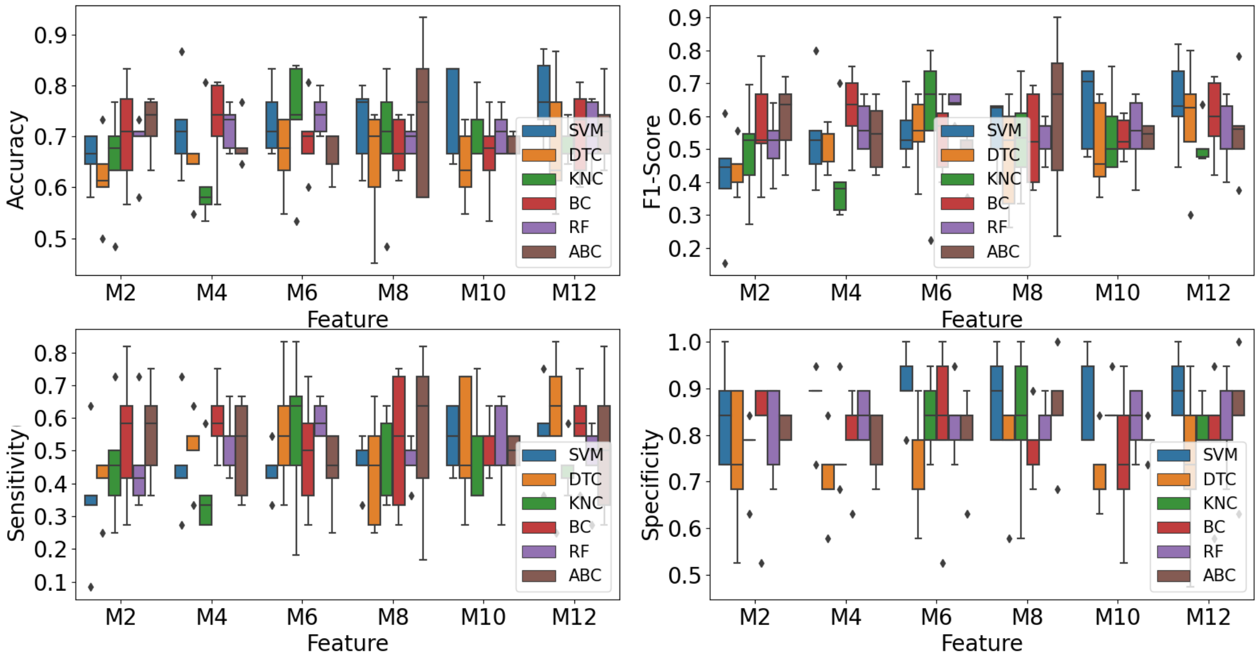

Figure 1: Classification results from 6 classifier. $M + number$ on the horizontal axis refers to how many microstates maps the features are derived from. For example, M4 refers to the features derived from 4 microstate maps.

5 Response to Review #3

Overall Comment: The paper presents detection of Ischemic Stroke through EEG Microstates. Major comments are

Comment 5.1: The rationale behind the usage of EEG must be clearly stated. Why not imaging techniques like CT, PET, or MRI which are more accurate? Also, the rationale and motivation of the study is not clear.

Our Response: *Thanks for your suggestion. Although the spatial resolution of EEG is lower than images such as CT, PET and MRI, EEG has its particular advantages. Firstly, EEG is suited to monitoring besides the bed for long-term and has higher time domain resolution. In addition, EEG is noninvasive and inexpensive compared with CT, PET and MRI. Therefore, EEG is commonly used for long-term monitoring of patients with brain injuries, especially in patients with disorders of consciousness. We have added two sentences to explain why we use EEG in the introduction section of the manuscript.*

> Although the spatial resolution of EEG is lower than images such as CT, PET and MRI, EEG is noninvasive, easy to monitor long-term and inexpensive, therefore, it is widely used in neurological examinations. Although the spatial resolution of EEG is not as good as images such as CT, PET and MRI, EEG recordings always have higher time domain resolution.

Comment 5.2: The literature review is still shallow. The authors are advised to add a table rather than reporting the existing work in text. this will make the manuscript more readable and easy to follow.

Our Response: *Thanks for your suggestion. We have added references [19, 20, 35, 36] in our manuscript to complete the literature. We did not provide a separate table to report the existing work as the separate table for reference may be a necessary part of a review paper. We introduce the existing work about EEG microstates in the 4th and show the potential of EEG microstates in monitoring the after-effects of stroke patients in the 5th paragraph in the introduction part of the manuscript.*

Comment 5.3: The authors are advised to perform 10-fold cross validation and LOSO. It would be a good test to check the stability of the performance.

Our Response: *Thanks for your suggestions. We have added cross-validation in our experiments. The additional part in the manuscript is as below:*

> *To ensure stable classification operations, we used the stratified 5-fold cross-validation method to train the existing classifiers. This method, widely used in classification, ensures that the proportions between classes are the same in each fold as they are in the whole dataset.*
> *The 5-fold cross-validation classification results from the existing classifiers are shown in Fig 8. From the figure, it can be seen that the mean accuracy and F1-Score from the six classifiers are all below 80%. Although the mean specificity is good, almost 90%, the means of sensitivity from all six classifiers are below 65%. Sensitivity representing how many subjects with DoC are predicted correctly is the most critical evaluation metric in this study.*

Comment 5.4: Why not use some preprocessing and feature extraction. In the current form, it is not clear how and what features are fed to the classifier.

Our Response: *Thanks for your suggestion. Our study has extracted EEG features to input to classifiers. The features we extracted are statistical parameters of microstates: duration, occurrence, coverage and GFP. These descriptions have been provided in the Classification Result Section of our manuscript as below:*

> *In our classification, the inputs to each classifier included four microstate parameters 'duration', 'occurrence', 'coverage' and 'GFP' as classification features from each microstate. The state of consciousness (DoC or wakefulness) was also inputted as a label to the classifier.*

In addition, the specific methods to extract the above EEG microstate features are provided in the Materials and Methods Section of our manuscript below:

> **Microstate parameters**
>
> *Microstate parameters, duration, occurrence, coverage, and variance were calculated in the following for the sorted microstates [9, 22]:*
>
> 1. *Duration: the average duration that a given microstate remains stable.*
> 2. *Occurrence: the number of times a microstate occurred during one second.*
> 3. *Coverage: the total percentage of time covered by a given microstate.*
> 4. *Variance: global explained variance (GEV) is a measure of how similar each EEG sample is to the microstate prototype that the sample has been assigned to [21]. The higher the GEV is, the higher the similarity is.*

Comment 5.5: The authors are advised to add confusion matrix with number of signals belonging to each class. It is unclear from the manuscript that how many signals are used for each class. Also, try to present the dataset details using table to have better readability.

Our Response: *Thanks for your critical comments and suggestions. In Fig 6, Fig 7, Fig 8 and Fig 9, we provided four valuation metrics: accuracy, sensitivity, specificity and F1-Score which are calculated from the confusion matrix. In our study, we use the above four metrics to demonstrate the classification performance from different classifiers and these metrics are the most common metrics to evaluate classifiers in machine learning. In addition, we provided the details of the dataset in Table 1, from the table, it can be seen that there are 95 negative subjects (awake patients) and 57 positive subjects (patients with disorders of consciousness).*

Comment 5.6: The authors are advised to compare the results with existing state of the art techniques.

Our Response: *Thanks for your suggestion. We have added experiments in the classification result section to compare our ensemble classifier EOSVM with six existing classifiers, including AdaBoost, Bagging, Random Forest, SVM, Decision Tree (DTC) and K-Neighbors (KNC). Compared with the existing classifiers, our EOSVM pays more attention to improving the predicting accuracy of the minor class in the dataset. The adding experiments results in the manuscript are as below:*

> **Classification Results from other existing classifiers**
>
> *To further explore the performance of existing classifiers on our dataset, we used the other four classifiers: DTC, KNC, BC, and RF, to classify subjects with DoC and without DoC.*

To ensure stable classification operations, we used the stratified 5-fold cross-validation method to train the existing classifiers. This method, widely used in classification, ensures that the proportions between classes are the same in each fold as they are in the whole dataset.

The 5-fold cross-validation classification results from the existing classifiers are shown in Figure 3. From the figure, it can be seen that the mean accuracy and F1-Score from the six classifiers are all below 80%. Although the mean specificity is good, almost 90%, the means of sensitivity from all six classifiers are below 65%. Sensitivity representing how many subjects with DoC are predicted correctly is the most critical evaluation metric in this study.

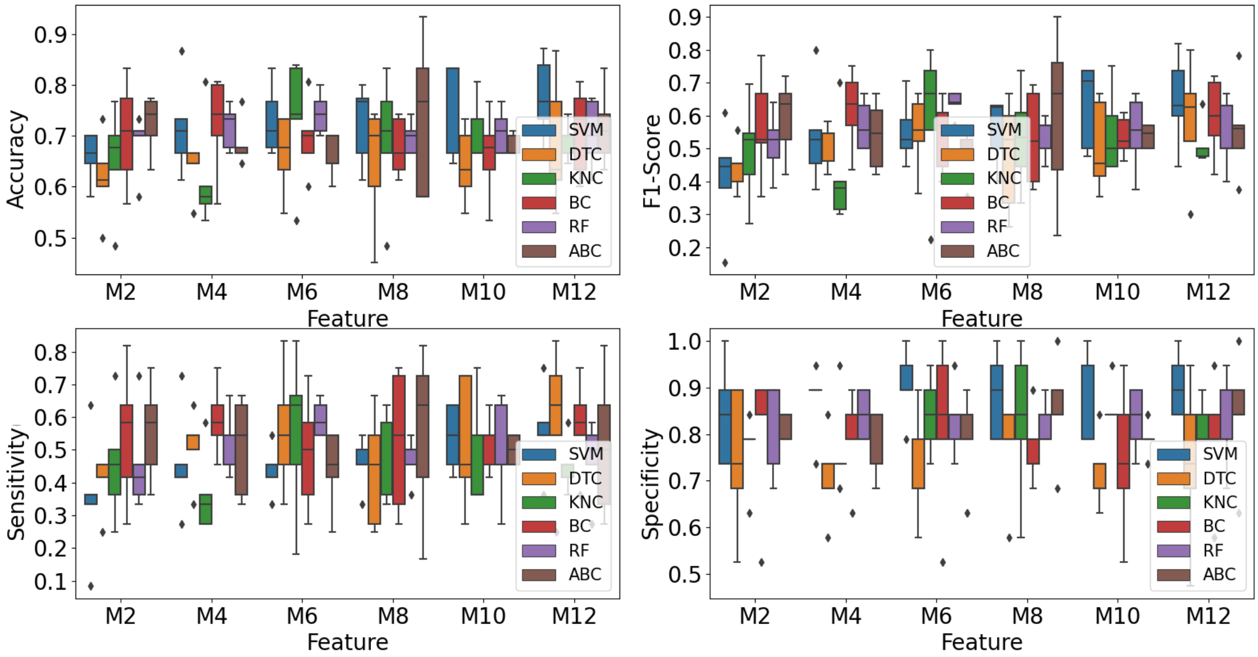

Figure 2: Classification results from 6 classifier. *M + number* on the horizontal axis refers to how many microstates maps the features are derived from. For example, M4 refers to the features derived from 4 microstate maps.

Comment 5.7: The results are poorly presented. The authors are advised to provide interpretation of the results. Also, can authors also provide discussion about the brain regions? Which channels perform better and what brain region has yielded the highest accuracy?

Our Response: *Thanks for your suggestions. As EEG microstates is a method that extracts features from the time dimension of EEG signals, this method focuses on the changes of signals in the time domain. In addition, our EEG dataset record signals from 8 electrodes which contain little space domain information. Compared with CT and MRI images, the advantage of EEG signals is monitoring the states of patients for a long time. The EEG spatial resolution is not high and provides little information in the space domain, especially for the EEG signals recorded from a small number of electrodes. Therefore, we did not provide a brain regions discussion.*

Comment 5.8: Try to compare the results with RF and other ensemble techniques to see if it achieves better performance than it.

Our Response: *Thanks for your suggestions. We have added the other four state of classifiers including K-Neighbors (KNC), Decision Tree (DTC), Random Forest (RF) and Bagging*

(BC) in our experiments.

Comment 5.9: Compare the results of classification with state of the art ML like KNN, decision tree, ANN.

Our Response: *Thanks for your suggestions. We have added the other four state of classifiers including K-Neighbors (KNC), Decision Tree (DTC), Random Forest (RF) and Bagging (BC) in our experiments. The additional subsection in our manuscript is as below:*

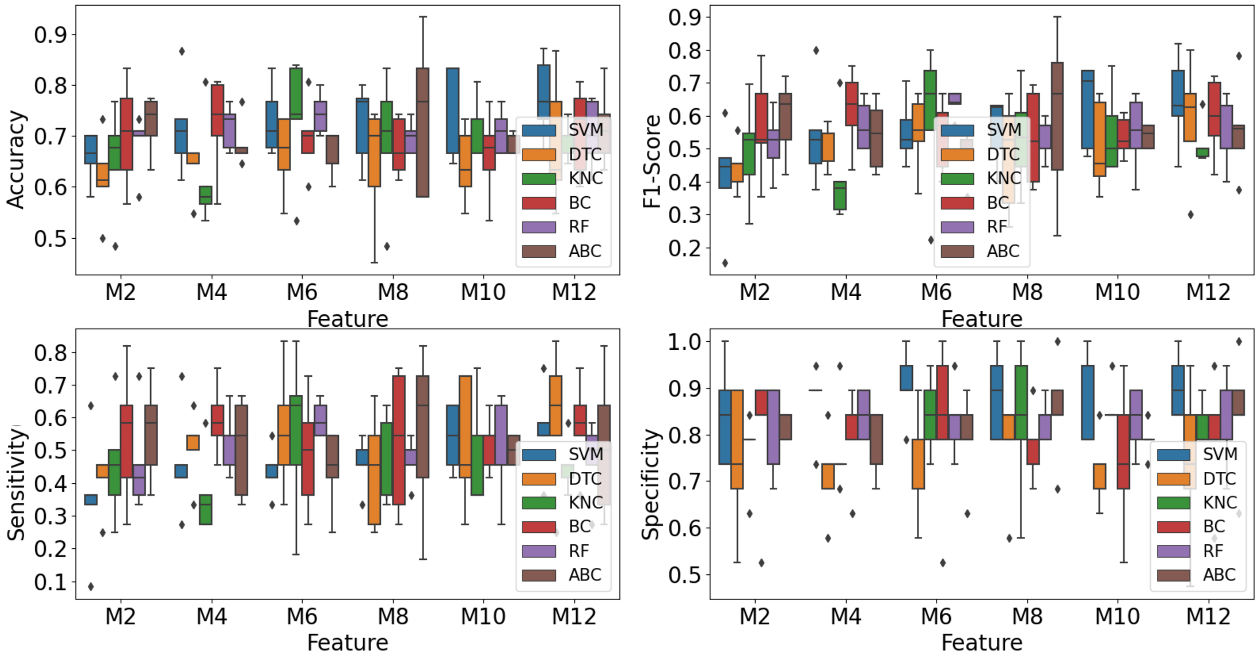

Figure 3: Classification results from 6 classifier. $M + number$ on the horizontal axis refers to how many microstates maps the features are derived from. For example, M4 refers to the features derived from 4 microstate maps.

Classification Results from other existing classifiers

To further explore the performance of existing classifiers on our dataset, we used the other four classifiers: DTC, KNC, BC, and RF, to classify subjects with DoC and without DoC.

To ensure stable classification operations, we used the stratified 5-fold cross-validation method to train the existing classifiers. This method, widely used in classification, ensures that the proportions between classes are the same in each fold as they are in the whole dataset.

The 5-fold cross-validation classification results from the existing classifiers are shown in Figure 3. From the figure, it can be seen that the mean accuracy and F1-Score from the six classifiers are all below 80%. Although the mean specificity is good, almost 90%, the means of sensitivity from all six classifiers are below 65%. Sensitivity representing how many subjects with DoC are predicted correctly is the most critical evaluation metric in this study.

Comment 5.10: Explore the uncertainty and explainability of the results. For e.g. cite https://www.preprints.org/manuscript/202301.0148/v1

https://www.sciencedirect.com/science/article/pii/S0010482523001415

Our Response: *Thanks for your suggestions. We have added several references [19, 20, 35, 36] in the current version of the manuscript.*

========END OF DOCUMENT========
