# Supplementary material for: Monitoring the after-effects of ischemic stroke through EEG microstates
Source: PLoS One. 2024 Mar 22;19(3):e0300806. doi: 10.1371/journal.pone.0300806 (PMC10959352; doi:10.1371/journal.pone.0300806)
Supplement: Attachment — Submitted filename: response_3.pdf [file pone.0300806.s003.pdf]

Response to the Review

Manuscript Ref No.: PONE-D-23-05197

"Monitoring the after-effects of Ischemic Stroke through EEG Microstates"

Fang Wang, Xue Yang, Xueying Zhang, Fengyun Hu

We extend our sincere gratitude to the editor, associate editor, and the anonymous reviewers for their invaluable comments and suggestions, which have significantly contributed to enhancing the quality and readability of our manuscript. In the previous iteration, reviewers 1 and 2 accepted our revisions and recommended it for publication. However, reviewer 3 provided critical feedback. We meticulously considered all comments and suggestions from reviewer 3 and implemented necessary revisions accordingly. Aligning with the Editor's guidance, we resubmit our revised manuscript for your further evaluation and consideration for publication.

We've provided comprehensive responses below to the editor and reviewers, delineating how we've addressed each of their comments and suggestions. In our revised manuscript, all modifications are highlighted in red for easy identification. These changes reflect our commitment to incorporating the insightful feedback received and refining our work to meet the standards for publication.

---

1 Response to Editor-in-Chief

a) The entire editorial decision letter:

PONE-D-23-05197R1 Monitoring the after-effects of Ischemic Stroke through EEG Microstates PLOS ONE

Dear Dr. Wang,

Thank you for submitting your manuscript to PLOS ONE. After careful consideration, we feel that it has merit but does not fully meet PLOS ONE's publication criteria as it currently stands. Therefore, we invite you to submit a revised version of the manuscript that addresses the points raised during the review process.

Please submit your revised manuscript by Aug 02 2023 11:59PM. If you will need more time than this to complete your revisions, please reply to this message or contact the journal office at plosone@plos.org. When you're ready to submit your revision, log on to https://www.editorialmanager.com and select the 'Submissions Needing Revision' folder to locate your manuscript file.

Please include the following items when submitting your revised manuscript: A rebuttal letter that responds to each point raised by the academic editor and reviewer(s). You should upload this letter as a separate file labeled 'Response to Reviewers'. A marked-up copy of your manuscript that highlights changes made to the original version. You should upload this as a separate file labeled 'Revised Manuscript with Track Changes'. An unmarked version of your revised paper without tracked changes. You should upload this as a separate file labeled 'Manuscript'. If you would like to make changes to your financial disclosure, please include your updated statement in your cover letter. Guidelines for resubmitting your figure files are available below the reviewer comments at the end of this letter.

If applicable, we recommend that you deposit your laboratory protocols in protocols.io to enhance the reproducibility of your results. Protocols.io assigns your protocol its own identifier (DOI) so that it can be cited independently in the future. For instructions see: https://journals.plos.org/plo guidelinesloc-laboratory-protocols. Additionally, PLOS ONE offers an option for publishing peer-reviewed Lab Protocol articles, which describe protocols hosted on protocols.io. Read more information on sharing protocols at https://plos.org/protocols?utm$_m edium = editorial - emailutm_s ource = authorlettersutm_c ampaign = protocols$.

We look forward to receiving your revised manuscript.

Kind regards,

Tommaso Martino, M.D. Academic Editor PLOS ONE

[Note: HTML markup is below. Please do not edit.]

Reviewers' comments:

Reviewer's Responses to Questions

Comments to the Author

1. If the authors have adequately addressed your comments raised in a previous round of review and you feel that this manuscript is now acceptable for publication, you may indicate that here to bypass the "Comments to the Author" section, enter your conflict of interest statement in the "Confidential to Editor" section, and submit your "Accept" recommendation.

Reviewer 2: All comments have been addressed

Reviewer 3: (No Response)

2. Is the manuscript technically sound, and do the data support the conclusions?

The manuscript must describe a technically sound piece of scientific research with data that supports the conclusions. Experiments must have been conducted rigorously, with appropriate controls, replication, and sample sizes. The conclusions must be drawn appropriately based on the data presented.

Reviewer 2: Yes

Reviewer 3: Partly

3. Has the statistical analysis been performed appropriately and rigorously?

Reviewer 2: Yes

Reviewer 3: No

4. Have the authors made all data underlying the findings in their manuscript fully available?

The PLOS Data policy requires authors to make all data underlying the findings described in their manuscript fully available without restriction, with rare exception (please refer to the Data Availability Statement in the manuscript PDF file). The data should be provided as part of the manuscript or its supporting information, or deposited to a public repository. For example, in addition to summary statistics, the data points behind means, medians and variance measures should be available. If there are restrictions on publicly sharing data—e.g. participant privacy or use of data from a third party—those must be specified.

Reviewer 2: Yes

Reviewer 3: No

5. Is the manuscript presented in an intelligible fashion and written in standard English?

PLOS ONE does not copyedit accepted manuscripts, so the language in submitted articles must be clear, correct, and unambiguous. Any typographical or grammatical errors should be corrected at revision, so please note any specific errors here.

Reviewer 2: Yes

Reviewer 3: No

6. Review Comments to the Author

Please use the space provided to explain your answers to the questions above. You may also include additional comments for the author, including concerns about dual publication, research ethics, or publication ethics. (Please upload your review as an attachment if it exceeds 20,000 characters)

7. PLOS authors have the option to publish the peer review history of their article (what does this mean?). If published, this will include your full peer review and any attached files.

If you choose "no", your identity will remain anonymous but your review may still be made public.

Do you want your identity to be public for this peer review? For information about this choice, including consent withdrawal, please see our Privacy Policy.

Reviewer 2: No

Reviewer 3: No

Reviewer 2: Thank you for your efforts on addressing all comments Your article is ready to get published in our journal

Reviewer 3: The authors did not answered all the comments. The authors failed to respond to almost 60% of the comments given in the previous version. The authors are invited to carefully go through the following comments and address it pointwise.

1. The authors have added the some lines about EEG and other modalities. Resting-state electroencephalography (EEG) monitoring provides an alternative way to potentially assist medical practitioners in a quick assessment of the after-effects of stroke. Although the spatial

resolution of EEG is lower than images such as CT, PET and MRI, EEG is noninvasive, easy to monitor long-term and inexpensive, therefore, it is widely used in neurological examinations. Although the spatial resolution of EEG is not as good as images such as CT, PET and MRI, EEG recordings always have higher time domain resolution.

But authors failed to cite any work. f the claims are not the authors original work, it is better to cite them. Please cite and follow the following articles strictly. The authors are recommended to provide wide acceptance of EEG due to their better temporal resolution. Like in drowsiness and emotion detection. In this line following papers are required to be cited:

https://www.sciencedirect.com/science/article/pii/S0003682X21002589

https://www.sciencedirect.com/science/article/pii/S0169260721005241

https://iopscience.iop.org/book/edit/978-0-7503-3279-8/chapter/bk978-0-7503-3279-8ch5

2. Ischemic Stroke: Few lines about this, their statistics around the globe will nicely introduce the problem and their seriousness. The authors are advised to add few statistics about the problem in the first paragraph of introduction.

3. The manuscript will be detailed, if it is compared with existing work on Ischemic Stroke. Try to locate some recent work and compare your results with it.

4. Confusion matrix: The reviewer is not satisfied with the explanation. Please provide the exact number in a Tabular form. The readers do not need to sit with calculator an evaluate the numbers from the Table 1. A separate Table of the actual numbers will make more impact on the manuscript.

5. A one good paragraph on Explainability and Uncertainty will provide readers with future direction. Please add few lines on it. Please cite the papers given in the last version as such works are on the rise because of scope in clinical settings.

https://www.preprints.org/manuscript/202301.0148/v

https://ietresearch.onlinelibrary.wiley.com/doi/full/10.1049/el.2020.2380

https://www.sciencedirect.com/science/article/pii/S0010482523001415

6. Try to strengthen the conclusion with your remarks, findings, and directions.

2 Response to Review #3

Overall Comment: Reviewer 3: The authors did not answered all the comments. The authors failed to respond to almost 60% of the comments given in the previous version. The authors are invited to carefully go through the following comments and address it pointwise.

Our Response: *We appreciate your feedback on our previous version. We acknowledge that we might not have fully addressed all the comments in the earlier version, and we sincerely apologize for this oversight. We have thoroughly reviewed all the comments and suggestions and are committed to addressing each point meticulously.*

Comment 2.1:

1. The authors have added the some lines about EEG and other modalities. Resting-state electroencephalography (EEG) monitoring provides an alternative way to potentially assist medical practitioners in a quick assessment of the after-effects of stroke. Although the spatial resolution of EEG is lower than images such as CT, PET and MRI, EEG is noninvasive, easy to monitor long-term and inexpensive, therefore, it is widely used in neurological examinations. Although the spatial resolution of EEG is not as good as images such as CT, PET and MRI, EEG recordings always have higher time domain resolution.

But authors failed to cite any work. f the claims are not the authors original work, it is better to cite them. Please cite and follow the following articles strictly. The authors are recommended to provide wide acceptance of EEG due to their better temporal resolution. Like in drowsiness and emotion detection. In this line following papers are required to be cited:

https://www.sciencedirect.com/science/article/pii/S0003682X21002589

https://www.sciencedirect.com/science/article/pii/S0169260721005241

https://iopscience.iop.org/book/edit/978-0-7503-3279-8/chapter/bk978-0-7503-3279-8ch5

Our Response:

We appreciate the reviewer's guidance and have revised the manuscript accordingly, integrating citations to substantiate the information provided about EEG's utility in stroke assessment. The revised manuscript is as below:

"Although the spatial resolution of EEG is not as good as images such as CT, PET and MRI, EEG recordings always have higher time domain resolution[6]."

Comment 2.2:

2. Ischemic Stroke: Few lines about this, their statistics around the globe will nicely introduce the problem and their seriousness. The authors are advised to add few statistics about the problem in the first paragraph of introduction.

Our Response:

We appreciate the valuable suggestion provided by the reviewer regarding the inclusion of statistics on Ischemic Stroke in the introductory section. In response to this advice, we have added the following setence in our Introduction section.

"The statistical data indicates that stroke ranks as the second leading cause of disability, con-

stituting 11.4% of disabilities among affected individuals, following closely behind dementia in the elderly population."

Comment 2.3:

3. The manuscript will be detailed, if it is compared with existing work on Ischemic Stroke. Try to locate some recent work and compare your results with it.

Our Response: *We acknowledge the reviewer's suggestion and would like to highlight that in the discussion section, we have extensively referenced and compared our findings with recent works related to Ischemic Stroke. The specific reference is [10,11,18,30,31,32,33,34].*

Comment 2.4:

4. Confusion matrix: The reviewer is not satisfied with the explanation. Please provide the exact number in a Tabular form. The readers do not need to sit with calculator an evaluate the numbers from the Table 1. A separate Table of the actual numbers will make more impact on the manuscript.

Our Response:

We appreciate the reviewer's feedback regarding the confusion matrix explanation. In response to this suggestion, we have included a separate table (Table 1) that presents the exact numerical values of the confusion matrix. We trust that this supplementary table offers a more impactful and straightforward depiction of the classification metrics. The table and the paragraph describles the table in the manuscript is as below:

Table 1: **Pearson Correlation Tabular Results.**

Pearson r	r	R squared	P (two-tailed)	P value summary	Significant? (alpha = 0.05)
L6 vs. Occurence_B	-0.2463	0.06068	0.0022	**	Yes
L6 vs. Occurence_C	-0.1792	0.03213	0.0271	*	Yes
L6 vs. Occurence_D	-0.3808	0.145	¡0.0001	****	Yes
L6 vs. Occurence_F	0.2198	0.0483	0.0065	**	Yes
L6 vs. Duration_A	0.4472	0.2	¡0.0001	****	Yes
L6 vs. Duration_B	0.2969	0.08817	0.0002	***	Yes
L6 vs. Duration_C	0.2227	0.04958	0.0058	**	Yes
L6 vs. Duration_F	0.1682	0.02828	0.0384	*	Yes
L6 vs. Coverage_A	0.189	0.03573	0.0197	*	Yes
L6 vs. Coverage_D	-0.2874	0.08259	0.0003	***	Yes
L6 vs. Coverage_F	0.1996	0.03985	0.0137	*	Yes

"Table 1 presents the results of Pearson correlation analysis corresponding to Fig. **??**, *outlining the correlation coefficients among 12 significant microstate parameters out of the total 18 features investigated. The table illustrates the Pearson correlation coefficient (r) values alongside their squared correlation (r squared), two-tailed p-values, and the summary of significance levels (using alpha = 0.05). Each correlation entry signifies the relationship strength between microstate parameters and the level of consciousness. Significant correlations, denoted by asterisks (*), ***, or ****, indicate the respective p-values being below the threshold of 0.05, highlighting statistically significant associations between these microstate parameters."*

Comment 2.5:

5. A one good paragraph on Explainability and Uncertainty will provide readers with future direction. Please add few lines on it. Please cite the papers given in the last version as such works are on the rise because of scope in clinical settings.

https://www.preprints.org/manuscript/202301.0148/v

https://ietresearch.onlinelibrary.wiley.com/doi/full/10.1049/el.2020.2380

https://www.sciencedirect.com/science/article/pii/S0010482523001415

Our Response: *We appreciate the reviewer's insight into the potential addition of a paragraph discussing Explainability and Uncertainty in the context of our study. However, after careful consideration, we find that incorporating this aspect might not align with the specific focus and scope of our research. While we acknowledge the relevance of works emphasizing Explainability and Uncertainty in clinical settings, we believe that the current manuscript extensively covers the primary objectives and findings related to stroke assessment using EEG signals. Given the specific nature of our investigation and its immediate clinical implications, we find that delving into these broader concepts might not complement the depth of our current study. We are thankful for the suggestion but feel that it might deviate from the core narrative and the emphasis of our research.*

Comment 2.6:

6. Try to strengthen the conclusion with your remarks, findings, and directions.

Our Response:

We appreciate the reviewer's suggestion to strengthen the conclusion further with remarks, findings, and future directions. Our study's conclusions highlight a significant relationship between EEG microstates and clinical states, notably consciousness and motor disturbances in stroke patients. Our statistical analysis revealed noteworthy correlations, notably the association between the occurrence of microstate A, duration of microstate D, and the sides of motor disturbance in stroke patients. Additionally, microstates A, B, C, D, and F demonstrated correlations with consciousness states across various parameters.

Furthermore, our classification approach employing an EOSVM classifier showcases promising results, indicating the potential for predicting DoC or wakefulness in stroke patients. With an accuracy exceeding 85%, predicting approximately 65.91% of stroke patients, our EOSVM-based classification using EEG microstates presents a viable, resource-efficient alternative to traditional clinical checks in DoC detection. These findings hold significant implications for medical practitioners, suggesting a potential avenue for utilizing EEG microstates as a valuable tool in DoC detection, ultimately conserving resources and streamlining clinical assessments. We believe that these conclusions underscore the practical utility of our research and provide directions for future investigations into leveraging EEG microstates for enhanced stroke patient care.

========END OF DOCUMENT========
